# DeepEdge: A Novel Appliance Identification Edge Platform for Data Gathering, Capturing and Labeling

**DOI:** 10.3390/s22072432

**Published:** 2022-03-22

**Authors:** Zilin Wang, Wei Wang, Ziyou Zhang, Fei Hu, Xingyi Xia, Liangyin Chen

**Affiliations:** 1 College of Computer Science, Sichuan University, Chengdu 610065, China; wangzilin@stu.scu.edu.cn (Z.W.); wang.david.wei@stu.scu.edu.cn (W.W.); 2020223045201@stu.scu.edu.cn (F.H.); 2020223045167@stu.scu.edu.cn (X.X.); 2 School of Control Engineering, Chengdu University of Information Technology, Chengdu 610225, China; 3 National Key Laboratory of Fundamental Science on Synthetic Vision, College of Computer Science, Sichuan University, Chengdu 610065, China; 2017326040007@stu.scu.edu.cn; 4 Institude for Industrial Internet Research, Sichuan University, Chengdu 610065, China

**Keywords:** load monitoring, tiny machine learning, Internet of Things, appliance identification

## Abstract

With the development of the Internet of Things for smart grid, the requirement for appliance monitoring has become an important topic. The first and most important step in appliance monitoring is to identify the type of appliance. Most of the existing appliance identification platforms are cloud based, thus they consume large computing resources and memory. Therefore, it is necessary to explore an edge identification platform with a low cost. In this work, a novel appliance identification edge platform for data gathering, capturing and labeling is proposed. Experiments show that this platform can achieve an average appliance identification accuracy of 98.5% and improve the accuracy of non-intrusive load disaggregation algorithms.

## 1. Introduction

With the development of the Internet of Things (IoT) and smart grid, the requirement for appliance monitoring has become an important topic [1]. The demand for high-quality electrical services has led to the increasing importance of electrical identification in recent years [2]. Energy consumption and management have attracted more attention due to rising energy prices and people’s increasing focus on environmental protection [3,4,5]. Most of the existing appliance identification platforms are cloud based, but they have some disadvantages such as great delays and a high cost [2]. At the same time, according to the characteristics of practical application scenarios, the appliance identification algorithm is implemented on the edge terminal to enhance the accuracy of the load disaggregation algorithm. Therefore, an appliance identification platform based on edge side has become a popular topic in academia and multiple industries [6].

Appliance monitoring methods are categorized into two classes, indirect monitoring [7] and direct monitoring. The indirect monitoring methods is also referred to as the non-intrusive load monitoring (NILM) algorithm. However, due to its complex mathematical model and inability to accurately identify the type of appliance, its accuracy is often not very high. Direct appliance monitoring methods can detect different types of appliances more precisely. The existing direct appliance monitoring and identification algorithms and systems are generally end-cloud collaborative ones which collect data from terminal sensors and process them in the cloud [8,9,10]. However, these systems have some disadvantages such as great delays and slow model update speed.

In order to address these issues, an experimental simulation system of the load disaggregation algorithm based on the appliance classification and identification algorithm is developed. It mainly includes the following content:

1. We developed an electrical identification terminal with edge intelligence. The hardware of the equipment terminal is divided into two parts: the first part is responsible for collecting the data of the high-frequency current transformer, while the second part is responsible for real-time calculations and collecting power consumption data. Through the communication pipeline of the IoT, the current waveform and power consumption data can be simultaneously gathered into the system of intelligent load monitoring energy. This can help to realize the intelligent management and data storage of appliances. It can also facilitate the data analysis and visualization in the subsequent research stage.
2. An intelligent load monitoring Internet of Energy (IoE) system with NILM capability is constructed. The IoE system is responsible for the identification of electrical labelled data, the model construction of the classification neural network, the reasoning of cloud classification, the construction of the software components of the edge terminal, and forming an experimental simulation system from the load disaggregation algorithm based on the appliance identification algorithm and cloud-side cooperation.
3. This experimental simulation system is based on the existing IoE users’ environment. It relies on multiple function smart meters and the smart strip sensor circuit, including hardware, software and an algorithm of minor changes. It collects energy data and mines the value of the IoE, so as to conserve energy, reduce emissions in users’ environment and ensure their security when using electricity.

The remainder of the paper is structured as follows: In Section 2, we present the works related to this topic. In Section 3, the general idea of this system is proposed in this work. In Section 4, we describe the specific design of the system. In Section 5, details of experimental setups, tests and accuracy results are presented. Finally, the work is concluded and possible future research directions are given in Section 6.

## 2. Related Works

Since Hart et al. first proposed NILM in 1992 [11], many studies have emerged in the field of NILM. In the beginning, NILM mainly used traditional machine learning, such as Hidden Markov Models [12]. In recent years, with the breakthrough of deep learning, neural network-based NILM methods have gained a lot of attention from researchers. Kelly et al. applied neural networks to load decomposition for the first time [13] and Zhang et al. used a simple sequence to point neural networks based on CNN [14]. Because of its high accuracy, it has become a benchmark algorithm in the field of deep learning for NILM. However, the high computation and memory requirements based on these approaches make it impossible for NILM to run in real time on low-cost microcontroller devices.

To make full use of edge devices, Aboulian et al. seamlessly combined wireless measurement devices that acquire local device current data with cloud services that run NILM algorithms to significantly improve the real-time performance of algorithms [15]. Chang et al. designed a load identification system based on the characteristics of residential electricity consumption and made it run in an Intel Atom processor [16]. Sirojan et al. implemented an embedded neural network for NILM by using the National Instruments (NI) myRIO-1900 platform containing a field programmable gate array (FPGA) and an ARM Cortex-A9 processor [17]. However, the chip running NILM algorithm in the above approach is still extremely costly compared to the embedded microcontroller. By using an Arduino microcontroller, Barsocchi et al. implemented a NILM platform capable of sampling low-frequency power data and identifying the sampled data on the microcontroller through a finite state machine [18]. However, this solution is not only less accurate, but also too large to be practically deployed. As shown in Table 1, none of the algorithms for load identification work perfectly on the edge side.

Our work is inspired by recent research findings. As discussed in [19], Powerblade has built a new, scalable, small-scale and intrusive load monitoring device for the purpose of load monitoring. This small terminal provides a new idea for the miniaturization of the digital signal processing algorithm. However, it does not design the model of the tiny machine learning algorithm and deploy it to this terminal, which uses the microcontroller as the core part. Zhang et al. realized neural network training and reasoning for keyword spotting in speech on low-end edge devices [20]. The system provides a theoretical basis and experimental exploration for the application of machine learning technology in low-end edge devices such as microcontrollers. Huang et al. implemented a gesture recognition algorithm and system based on the RNN algorithm under optical conditions. This system provides theoretical support and experimental research for neural network technology to solve the problem of high-frequency timing sequence data analysis in the edge terminal [21]. Through conducting a literature review, it was found that the appliance identification algorithm on the terminal is a new way to improve the performance of the NILM algorithm. At the same time, the EdgeImpulse platform was discovered by adopting existing machine learning experimental simulation systems [22]. It is suitable for time series data acquisition and label engineering in processing edge machine learning algorithm construction. Therefore, this work constructs an experimental simulation system of load disaggregation based on the appliance identification algorithm. It implements some relevant algorithms on edge equipment, which improves the accuracy of the appliance identification algorithm and the load disaggregation algorithm.

## 3. The General Idea of the System

**Figure 1:**
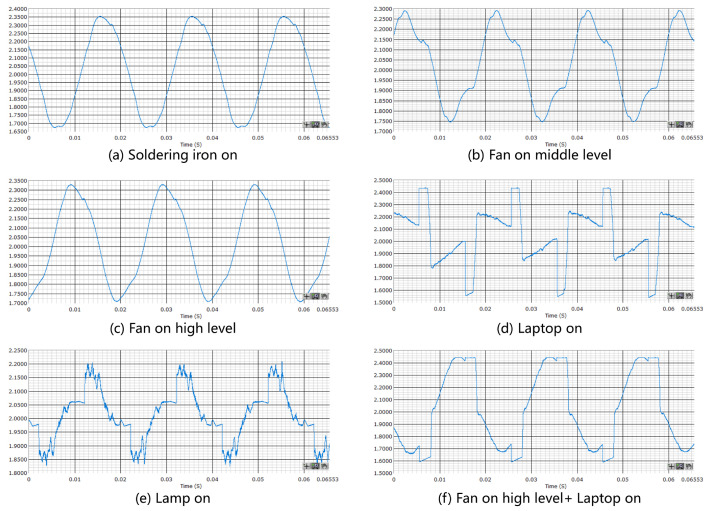
Different current waveforms for different appliances sampled at 1 MHz.

After analyzing the literature, the research motivation for constructing the experimental simulation system is further verified by these experiments. In order to quickly complete our research, the virtual experimental simulation system was constructed through the programmable dynamic high-precision analog to digital conversion chip and the digital signal processing module. Then the following experimental results were obtained. The experimental results in Figure 1 show the current waveform curves of different appliances sampled at 1 Mhz. It can be seen that there are obvious differences in these current waveforms. Since the soldering iron is a purely resistive load, its current is a sine wave, shown in Figure 1a. Similarly, as the power of the electric fan rises, shown in Figure 1b,c, its capacitance becomes more and more pronounced and gradually changes to a sine wave. The laptop computer shown in Figure 1d, shows a sawtooth wave due to the presence of a switching power supply for AC-DC conversion, and the electric lamp shown in Figure 1e shows a sine wave with a burr because it contains both a switching power supply and a resistive load, while in the case of multiple appliances shown in Figure 1f, a more complex current pattern emerges. Furthermore, our work considers the relationship between sampling overhead and classification accuracy. It aims to conduct experiments at sampling frequency rates of 10 khz, 20 khz, 50 khz and 100 khz, respectively. We also aim to measure and record sensor waveform curves to verify the research motivation for this work.

Due to the difficulty of collecting labeled data samples from the IoE [23], the method provided by the sequential data annotation platform solves the problem of collecting data sets and realizing the labeled data required for appliance identification. It stores them in the cloud for use in subsequent training and reasoning stages. According to the Shannon sampling theorem [24], the physical signal should be sampled with a sampling frequency twice that of the feature to be extracted in order to retain the high-frequency characteristics of appliances. As a new type of signal processing algorithm, the neural network model is essentially used to achieve the parameter fitting of the mapping function, and it also conforms to the Shannon sampling theorem.

For the neural network model trained in the cloud, the distribution mechanism of the microcontroller software interface standard is adopted to generate software components that correpond to the neural network model. Embedded program codes are generated with the help of special tools and deployed to the microcontroller of the load sensor. This is the first time to realize the edge terminal of the appliance identification algorithm for the load disaggregation algorithm. It improved computational speed, so the time cost of the appliance identification algorithm reduced from a second to a millisecond.

An experimental simulation system of the load disaggregation algorithm based on the electrical recognition algorithm was constructed. We obtained high frequency and low frequency load data. It integrates the latest edge computing theory, embedded technology and signal processing machine learning algorithm, providing great convenience for the load analysis research group and research community.

**Figure 2:**
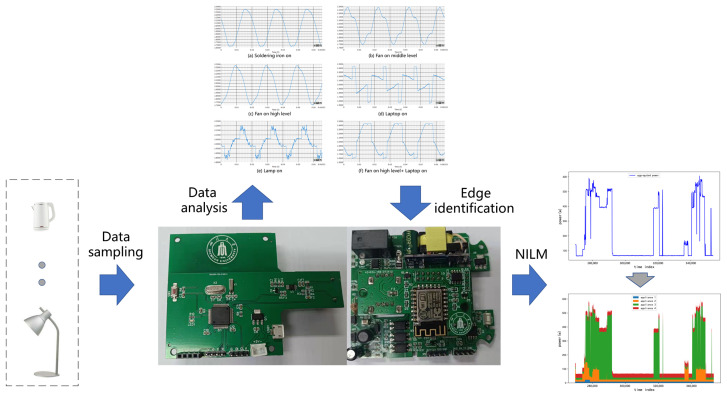
Diagram of appliance identification and NILM in a mixed load monitoring scheme.

Using a software method and reducing the cost of sensor network hardware for power monitoring have become the true value and practical significance of NILM algorithm research. Therefore, the research and system construction of these kind of algorithms have become a research hotspot in the field of IoE in recent years [25]. In our work, an experimental simulation system of the load disaggregation algorithm based on appliance recognition was constructed, as shown in Figure 2. The low-cost hardware of deepEdge can collect current and power data from appliances with a high sampling rate, and upload them to the cloud for data analysis and model training. After that, the model is downloaded back to the hardware for edge-side appliance type identification, and finally, by using the identification results of deepEdge, the NILM algorithm obtains better decomposition accuracy. The work of the simulation experiment system is divided into the following steps. Firstly, by optimizing the circuit design, the signal conditioning of the current transformer and power sensor on the sensor network node is realized. Secondly, data collection and data label engineering are performed. Then, the tiny machine learning model is constructed, trained and deployed. Next, the sensor code generation, code off-line compilation and firmware update are realized. Finally, the algorithm of the machine learning system is developed and applied. In addition, it also realizes the visualization of the result of the appliance identification and load disaggregation algorithm.

The power consumption data of electricity meters mainly come from public datasets, such as AMPds [26], REDD [27] and UK-DALE [28], while the power consumption data of actual experimental simulation systems can come from actual sensors. In the environment of IoE users, for the convenience of experimental simulation, we present a set of the truly developed IoT terminal and management system, which is used to collect the data of the current transformer and power sensor in real time for data storage, data sorting and data analysis.

After the completion of the above load experiment, we demonstrate the appliances that the high-frequency data can be used to identify. In order to further validate the feasibility of the electrical load disaggregation algorithm based on appliance identification, we built a close to the actual deployment experiment simulation system, which includes the sampling circuit and current sensor used in the electric power sensor circuit for data collection and storage of the IoT cloud platform. It is used for algorithm analysis of the backend management system and is a visual interface for displaying laboratory simulation results.

In our work, an experimental simulation system was designed to realize the appliance recognition algorithm on the edge terminal and to study the NILM algorithm. The specific engineering implementation steps are as follows: First of all, the current data of the main current loop are induced by the deployment of the precision current transformer, and the current waveform curve of the aggregation power consumption collection point is collected to realize the original collection of current data. At the same time, the power consumption data are collected by the HLW8032 chip. In the second step, the analog signal conditioning circuit is used to convert the current induced by the transformer into voltage signals, and the voltage follower and signal amplification are realized through the operational amplifier chip. Then the built-in high-speed analog-to-digital conversion chip is used to realize the real-time conversion of high-frequency current signal data. In the third step, the communication protocol that meets the requirements of the EdgeImpulse platform is realized and the power consumption data are uploaded through the program design of the micro-controller. The fourth step entails using the EdgeImpulse platform to carry out the tedious process of testing electrical events, collecting data and tagging data. It is more efficient than traditional methods though it is relatively heavy. According to the characteristics of high frequency data, the rules of cloud signal processing and the model of neural network are adjusted. In the fifth step, according to the collected electrical event data set, the data set is divided into two parts, namely, a training data set and a test data set. The classification rule is generally 80% training set and 20% test set [6]. The sixth step entails carrying out specific data feature engineering and training the neural network model with data. In the seventh step, the trained model is deployed on edge devices. First, the EdgeImpulse platform generates the distribution package that meets the microcontroller software interface standard. Then, the distribution package of the microcontroller is converted into code and the binary file that can run on edge devices is generated by the compiler.

## 4. Specific Design of the deepEdge Experimental Simulation System

**Figure 3:**
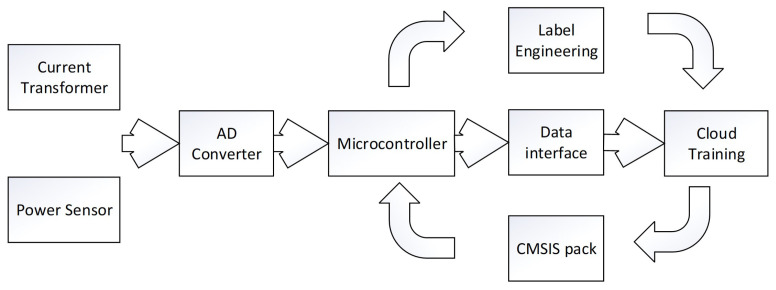
The procedure of implementing the deepEdge appliance identification algorithm.

After completing the overall design, the specific design of the experimental simulation system is described, including sensor circuit conditioning, data acquisition tag engineering, **deep** neural network training and testing, **edge** deployment, cloud system visualization and other links. As shown in Figure 3, in order to identify appliances, the simulation system of this experiment collects current data as the sensor input of the appliance identification algorithm. The specific work includes accessing data from the high-frequency current acquisition circuit, collection and classification of labeled sample data, and establishment of the signal processing algorithm module and machine learning algorithm model. Based on the tag sample data collected, the data features are generated, and the parameters of the training machine learning model are trained with the data features. The parameters of the training model are used to deduce the electrical event types. To obtain the load disaggregation algorithm, the experimental simulation system of power consumption data, a disaggregation algorithm for load sensor input, specific work including Internet access, and low power consumption data are used to calculate the method of environmental load disaggregation. According to the classification results of the appliance identification algorithm, combined with the load disaggregation algorithm, comprehensive results of the negative charge disaggregation algorithm are obtained.

### 4.1. Sensor Circuit

The circuit conditioning part of the sensor is divided into two parts. One is the signal conditioning of the sampling circuit of the precision current transformer, which is used to realize the real-time acquisition and conditioning of the ac electrical signals in the main circuit. The other part is the tuning part of the power sensor circuit, which is used to accurately measure the real-time power consumption data of the load. The two sub-parts constitute the sensor circuit of the load disaggregation algorithm experimental simulation system based on the electrical identification algorithm. They are used for the numerical conversion of pressure signals. An operational amplifier AD8552 is used to adjust the input voltage range from weak voltage to microcontroller. The operational amplifier AD8552 contains two operational amplifier units: one is used to follow the output voltage of the TL431 chip to achieve stable 1.5 V voltage output, and the other amplifier unit is used to achieve voltage amplification, which is zero. Specifically, the TL431 chip samples the entire current. The circuit and conditioning circuit provide a steady supply of voltage sources. The K-pole output of the TL431 chip is 2.5 V, and the stable 1.5 V reference voltage output is achieved through the partial voltage conversion of Equation (1).

(1) $$VCC\_1.5\;V=\frac{1\;k\Omega }{1\;k\Omega +1.5\;k\Omega }*2.5\;V$$

#### 4.1.1. Current Transformer Circuit

**Figure 4:**
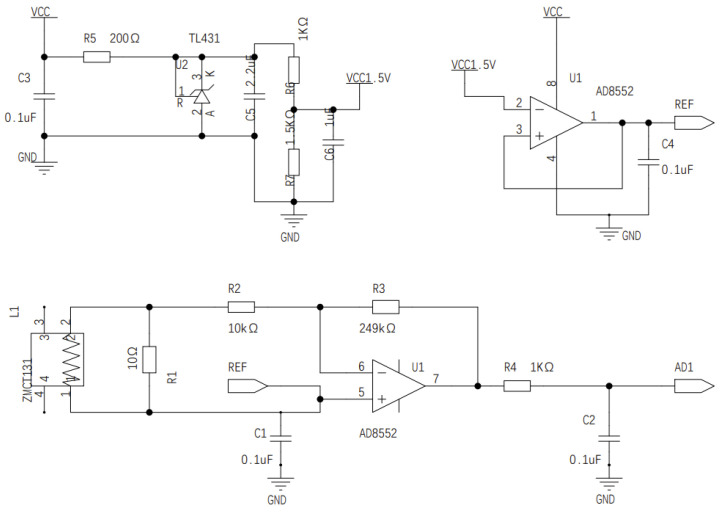
Current sampling and signal conditioning circuit based on the current transformer.

As shown in Figure 4, the analog front end of the current sampling circuit uses the precision current transformer made by Nanjing Zeming Company. The model is ZEMCT131, and the parameter is 5 A/2.5 mA variable ratio (i.e., 2000:1). Since the switching events of electrical appliances produce small current changes, corresponding signal conversion circuits need to be designed to facilitate the subsequent design of signal processing algorithms and machine learning algorithms. Firstly, the instantaneous current signal and electricity of the sampling loop are realized through the precision resistor R1 = 10.

Where, *V* is the input circuit voltage from the analog to digital converter, and *I* is the current of the AC circuit. According to Equation (2), the value of *V* is 0.125i. As the reference voltage of the operational amplifier is 1.5 V, the voltage range of the microcontroller is 0–3.3 V, and *V* is 1.5 V, the $I_{\mathrm{main}}$ is 12 A.

(2) $$V=\frac{I_{\mathrm{main}}}{2000}*10*\frac{249\;k\Omega +10\;k\Omega }{10\;k\Omega }$$

The driving ability of the output voltage of the reference source is insufficient; therefore, an operational amplifier may be increased to build the electric voltage follower and increase the current driving ability for the reference voltage to be stable and consistent. The following formula will drive access to the microcontroller of the analog signal input pin.

Considering that the ac weak current will fluctuate around the zero value after the ratio is changed, for the convenience of sampling, the current is converted to the voltage signal, and the value is translated to 1.5 V through the calibration voltage source of the circuit (equivalent to the median value of the full range voltage of 3.3 V). Therefore, the numerical transformation of the total current can be expressed by Equation (3).

(3) $$V_{\mathrm{AD}1}=1.5\;V+\frac{I_{\mathrm{main}}}{2000}*10*\frac{249\;k\Omega +10\;k\Omega }{10\;k\Omega }$$

**Figure 5:**
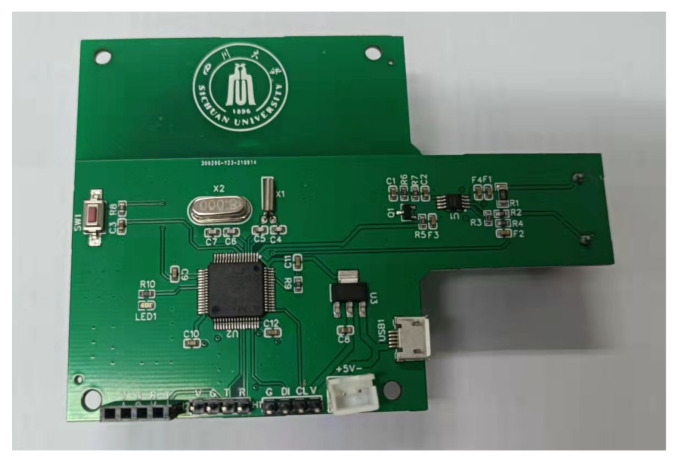
The circuit board of current sampling and signal conditioning based on the current transformer.

Figure 5 shows the circuit board of the current sampling signal conditioning circuit. There is a current transformer in the figure, and the measuring part is divided into a perforated structure, which does not produce insertion loss and has strong overload capacity, and forms a good electrical isolation from the strong electric part. Between the current transformer and the main chip, the sensor circuit conditioning part was designed. In order to collect data, a wireless data transmission module was designed below the transformer, which can transform data into a time series data labeling system.

#### 4.1.2. Power Consumption Transformer Circuit

**Figure 6:**
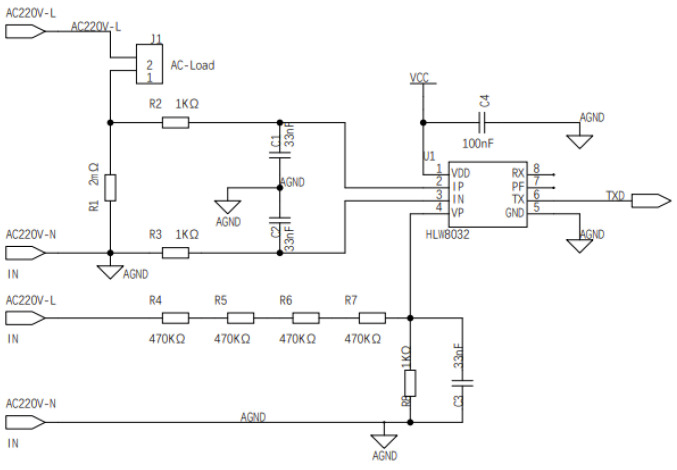
The power consumption calculation and acquisition circuit.

**Figure 7:**
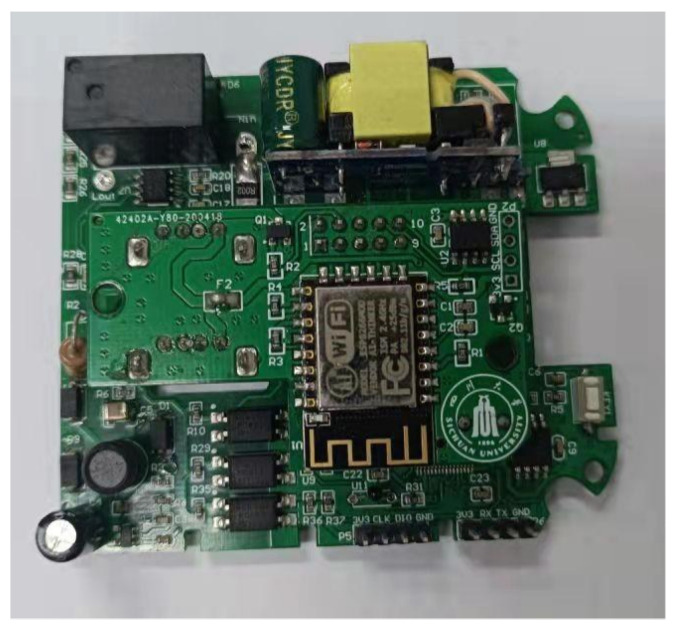
The circuit board of the power consumption calculation and acquisition circuit.

The important research task of our work is to realize the load disaggregation algorithm, so the load power consumption data are the most important sensor data. Because of the inconsistencies caused by circuit board manufacturing, software configuration is often required to carry out tedious circuit coefficient correction work in order to achieve the accurate detection of power consumption data. Therefore, in order to reduce inconsistency and provide consistent and reliable power consumption values for subsequent digital signal processing and the machine learning algorithm, the power consumption monitoring chip HLW8032 was adopted as the core chip of the load power monitoring circuit, as shown in Figure 6 and Figure 7. HLW8032 is a single-phase electric energy calculation chip, which has the ability of non-calibration, so it has been widely used in product design fields such as for the smart electricity meter and smart plug. The chip peripheral includes analog voltage chip power supply, sensor analog input, including voltage, current and numerical output, it also includes serial port data and high-frequency pulse data output. Due to the low frequency sampling data output, the output voltage and current of the circuit are both effective. The specific formulas are shown in Equations (4) and (5).

(4) $$V_{RMS}=\frac{{\mathrm{Reg}}_{v_{\mathrm{param}\;}}}{{\mathrm{Reg}}_{v}}*{\mathrm{Coeff}}_{v}$$

(5) $${\mathrm{Coeff}}_{v}=\frac{470k*4}{1k}*1000=\frac{1880k}{1000k}=188$$

(6) $$I_{RMS}=\frac{{\mathrm{Reg}}_{i_{pram}}}{{\mathrm{Reg}}_{i}}*{\mathrm{Coeff}}_{i}$$

(7) $${\mathrm{Coeff}}_{i}=\frac{1}{R}*1000=\frac{1}{0.001}*1000=1$$

(8) $$P=\frac{{\mathrm{Reg}}_{P_{\mathrm{paran}\;}}}{{\mathrm{Reg}}_{P}}*\;{\mathrm{Coeff}}_{v}*{\mathrm{Coeff}}_{i}$$

(9) $$S=V_{RMS}*I_{RMS}$$

(10) $$\cos\Phi =\frac{P}{S}$$

The power consumption chip can calculate effective voltage, effective current, active power, reactive power rate and power factor by the above Equations (6)–(10). Then, the chip is sent to the serial port at a rate of 4800 baud, in the format of eight data bits, one parity bit, one stop bit, 50 ms sending time interval per second. The microcontroller receiving data periodically receives the power data from the power sampling chip through the software design, so as to realize the real-time acquisition of low-power data.

### 4.2. Data Collection and Label Engineering

The data acquisition and label engineering of the system is described from three aspects: the software distribution mechanism of the microcontroller software interface standard, the analog data processing process based on the sensor circuit, and sample data labeling based on EdgeImpulse.

#### 4.2.1. Software Distribution Mechanism Based on Microcontroller Software Interface Standards

Cortex Microcontroller Software Interface Standard (CMSIS) is an embedded application Software framework provided by ARM. To better implement software distribution, the company developed a software distribution mechanism based on the microcontroller software interface standard, the CMSIS-Pack distribution. The distribution mechanism can implement the distribution of software components, device parameters and circuit board support. An XML-based package description file describes several broad sections, such as source code, documentation, sample code, device parameters, startup algorithms, programming algorithms, and sample projects. We relied on the generation tool of the system and used the trained neural network model parameters to generate the software package conforming to the microcontroller software interface standard distribution mechanism.

#### 4.2.2. Analog Data Processing Link Based on the Sensor Circuit

The signal conditioning circuit collects the original data and pushes them to the software platform EdgeImpulse to realize the fragmented sampling of current data and power consumption data, and to classify and store the data. To facilitate data collection, the script tool is used from the serial port to the cloud center and the baud rate is set to 115,200 BPS. The data rate reaches 115,200/(1 + 8)/1024 = 12.5 K bytes per second. Based on the communication protocol designed by this system, the floating point number format is 4 bytes, for example, “1.1234+ Carriage return newline”. This shows that the current sampling analog data are 1.1234 V. In this way, 3.125 k samples per second can be achieved. In order to achieve a higher frequency sampling rate, we used the design of the microcontroller model STM32L431RCT6 chip of STMICROELECTRONICS. The design selects one of the Analog to Digital Converter (ADC) channels to realize fast Direct Memory Access (DMA) analog data transfer and fast analog-to-digital conversion function.

#### 4.2.3. Labeling of the Sample Data Based on Edgeimpulse

**Figure 8:**
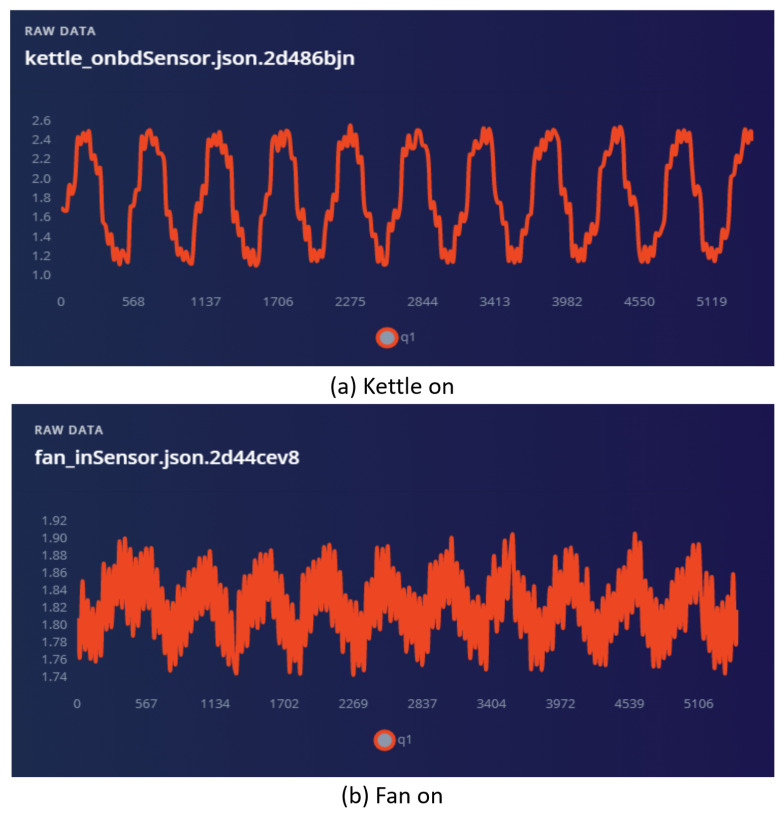
The current waveform of kettle on and fan on.

Real-time data are pushed to the cloud system through the edge-impulse-data-forwarder command line tool provided by the system. As shown in Figure 8, the sampling circuit obtains 6000 ms of data at a frequency of 912 Hz and collects analog data from the load sensor. First, we opened the kettle to acquire a current waveform of a length of 6000 ms, marked as ’Kettle_on’ label. Similarly, training data sets and test data sets were collected. Then, we repeated the above data collection and data annotation many times to complete the data set.

In analyzing the current waveform of the fan starting, it can be seen that the current waveform of the fan starting and the current waveform of the kettle turning on form an obvious contrast. Therefore, it is possible to improve the accuracy of the load disaggregation algorithm by collecting tag engineering of high-frequency current data and implementing the electrical identification algorithm. Through the software framework based on the microcontroller software interface, analog data processing based on the sensor circuit, and sample data labeling based on EdgeImpulse, we completed the data preparation for the neural network.

### 4.3. Deep Neural Network Construction Training Test

The experimental simulation system designed based on the completion of electricity-related data collection and label samples, continues to iterate the models and parameters suitable for the actual scene through the design model, training model and test model. The algorithm model construction includes a signal processing module, a data preprocessing module and neural network model construction. In the hybrid energy monitoring network, both current and power data are time series data, and fast Fourier transform and numerical filtering can be added to the signal processing part to improve the efficiency of data processing. Currently, there are three types of end-to-end deep neural networks, namely, fully connected neural networks (FNN), convolutional neural networks (CNN) and recurrent neural networks (RNN) represented by LSTM. For the LSTM network, since one neural unit has four gates, it is four times the size, thus it is not suitable for use in a microcontroller. In terms of classification performance, the work of Zhang et al [14]. shows that a one-dimensional CNN network has higher accuracy than a fully connected network, therefore, in this work, we chose 1D CNN to realize the function mapping of input data and output features.

**Figure 9:**
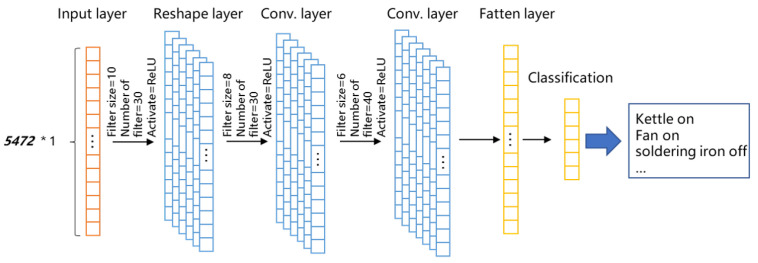
The model construction of the neural network.

As shown in Figure 9, in order to subsequently deploy the neural network model into the microcontroller and finally realize the deployment of the edge device side, we selected a relatively small neural network model. For example, in the neural network model in this figure, the input layer is the features formed by 5472 dimensional data, the output layer is the electrical event types to be classified, and the output feature is the total number of electrical events. In addition, the neural network contains six hidden layers. The first layer is the reshape layer, the second layer is 1D convolution layer, the third layer is the Dropout layer, the fourth layer is the 1D convolution layer, the fifth layer is the 1D convolution layer and the sixth layer is the flatten layer. The learning rate was set to $5\times 10^{-4}$, and Adam was used as the optimizer of the neural network.

As the sampling frequency mentioned above is 912 Hz, the input data feature of the classification neural network is 912 ∗ 6 s = 5472 dimensions, and the output feature is the total number of label types. Macroscopically, the purpose of the model is to input 5472 dimensions of multiple label samples and output data as the number of label types for continuous training of the model designed in our work, and to mine electrical appliance types and operation rules from the data samples. According to the experimental results, the 5472 features of the input layer can be achieved through the signal processing module to reduce the dimension of the data features, accelerate the realization of the classification function, for fast electrical appliances identification.

Embedded machine learning algorithm is a new research field, and the latest research achievement is the running of a machine learning algorithm in a microcontroller chip. As the core chip of the smart electricity meter, this kind of microcontroller mainly realizes the data acquisition, calculation, storage and communication functions of the smart electricity meter or smart plug and strip. The electric appliance identification and NILM algorithm realized in our work, especially the electric appliance identification algorithm realized by edge equipment, will have a far-reaching influence on the development of IoE.

### 4.4. Edge Deployment

There has been a considerable amount of research on appliance identification as evidenced by reading the current and power consumption characteristics of appliances. However, most of these studies use directly purchased sensors to collect appliance data and upload it to the cloud with more computing power for processing, while in the DeepEdge system, its model for identification sensor data runs directly in the microcontroller instead of uploading it. By this approach, we make full use of the computing power of the edge-side devices, which significantly decreases the recognition time of the appliance data. The specific deployment process is as follows. Firstly, through the sensor circuit conditioning part, the data acquisition tag engineering part and the model construction training and testing part, the neural network model and parameters that can be used for edge deployment with electrical equipment identification ability are obtained in theory. Secondly, a deployable neural network model is constructed through the EdgeImpulse platform, it is transformed into a software distribution package conforming to the microcontroller software interface standard and loaded into the data processing code engineering of the sensor to generate an embedded code that can be compiled and deployed. The code can then be loaded into the compilation environment of the sensor device to generate a binary code that can drive the device to properly implement data acquisition and model edge reasoning. After the training and testing described in the previous section and several iterations, the mold parameters will achieve a performance acceptable for users or researchers. Finally, the PowerStrip.1.0.10. Pack distribution for embedded development was released as a microcontroller software interface standard. Powerstrip is the project name, and 1.0.10 is the model version number. The reasoning result of the deployment scheme realized by the above method shows that the electrical equipment identification function can be realized quickly and accurately. The inference time is 136 ms, the required memory space is 87.6 K bytes, and the required code space is 74.2 K bytes. Therefore, it can be seen that the electrical recognition not only has high accuracy, but also has a fast inference time, and the memory and code space occupied can be fully operated in the current embedded system.

### 4.5. Cloud Visualization

**Figure 10:**
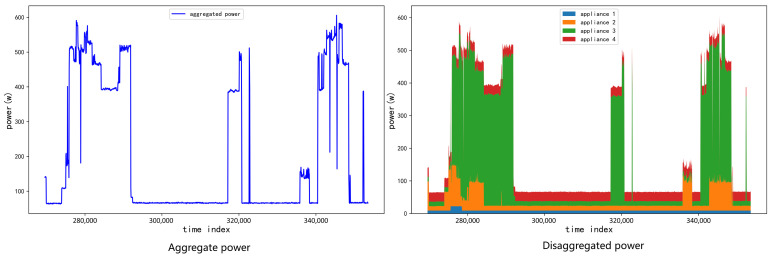
NILM results with the assistance of appliance identification.

The cloud design of the experimental simulation system includes data acquisition, equipment management, data processing and background design of algorithm design. JupyterLab tuning method was used for the continuous iteration of the electrical identification and load disaggregation algorithms. At the same time, in order to continuously sample high frequency and low frequency data, JupyterLab is used to obtain high frequency data to realize the electrical identification function, the pre-operation of low frequency data load disaggregation, and enhance the final effect of load disaggregation. As shown in Figure 10, based on the electrical equipment identification algorithm of high-frequency data, the real deployment of the NILM algorithm is realized in combination with the smart plug data. Different color areas in the figure represent the power values of different electrical appliances.

## 5. Analysis Results of the Experimental Data

Compared with the data collected by the advanced digital signal processing device in Figure 1, the data collected by the hardware device we designed, shown in Figure 8, has achieved comparable results. As shown in Table 2, the experimental setting is that the current sampling rate is 50 khz, which means that 50,000 samples of 12-bit analog-to-digital conversion resolution are achieved per second. Since the frequency of the AC current waveform is 50 Hz in China, the fundamental wave period is 20 ms. In order to capture multiple fundamental wave periods, the sampling range is selected to cover five times the fundamental wave periods, namely 100 ms time length. Therefore, it can be known that the number of 100 ms is 5000 sampling points at 50 khz sample frequency. Through the experiment, it takes about 6 s to convert data according to the protocol and transfer them to the cloud through websocket tool with 115,200 baud rate of serial port tool. In order to transmit the current waveform with sampling frequency of 50 khz, 100 ms sampling data can be transmitted within 6 s under the condition of ensuring the highest utilization rate of serial port data. As a result, the sampling content has been delayed by 5.9 s. Although the current waveform feature with a sampling rate of 100 khz is optimal, in order to give consideration to time delays and accuracy, the sampling rate was set to 50 khz. For the convenience of the experiment, four electrical appliances, namely a kettle, fan, lamp, and laptop, were selected as the research objects of the experimental electrical appliances, and the related experimental results were drawn. As shown in Table 3, the identification accuracy of all electrical appliances turned off was 100%, the accuracy of the electric iron turned on was 97.1%, and the kettle turned on was positive. The accuracy rate was 100%, compared with 51% for the laptop, and 49% for the kettle. The identification accuracy for appliances using a high-frequency power current waveform is above 90%. At the same time, the low-frequency power consumption data of the smart plug can be obtained, then the error of the load disaggregation algorithm can be calculated in real time. On the basis of the assistance of electrical identification, the accuracy of load disaggregation can be improved.

## 6. Conclusions

In our work, we first analyzed the weaknesses of two existing load monitoring algorithms, and in order to solve these weaknesses, we desigedn and implemented an appliance identification system including hardware, software and algorithm design, which cannot only identify appliance types, but also mark high frequency appliance current/power consumption data and store them in the cloud. Furthermore, we designed a load monitoring system with load decomposition capability, which can fully utilize the recognition capability of the appliance identification system to conform to the total power consumption decomposition, thus achieving a NILM effect with high accuracy. Finally, we conducted experiments on both systems, where the end-to-end neural network achieved high recognition accuracy in the appliance recognition capability, and the accurate appliance recognition capability improved the NILM by more than 10% in the load decomposition experiment.

In the future, we will coordinate the computational performance on both the cloud and the device side through dynamic computing power scheduling to maximize the accuracy of the load decomposition algorithm. In addition, we will further optimize the architecture of the system so that it has excellent expansibility to provide more powerful software, hardware and algorithm support for researchers in the load disaggregation research community.

## Figures and Tables

**Table 1:** Disadvantages of existing solutions.

Research	Running Environment	Disadvantage
Aboulian et.al.	High performance PC	The edge side only collects power data and does not run algorithms
Chang et al.	Intel Atom processor	High cost of edge-side chips
Sirojan et al.	ARM Cortex-A9 processor	High cost of edge-side chips
Barsocchi et al.	Arduino	The edge side’s size is large, and the identification accuracy is low

**Table 2:** Sampling rate and accuracy of appliance identification.

Sampling Rate (khz)	Number of Samples	Transmission Time (s)	Accuracy
10	1000	1	low
25	2500	2.5	low
50	5000	6	high
100	10,000	12	high

**Table 3:** Confusion matrix of appliance identification.

	All off	Soldering Iron on	Kettle on	Laptop on
All off	100%	0%	0%	0%
Soldering iron on	2.9%	97.1%	0%	0%
Kettle on	0%	0%	100%	0%
Laptop on	0%	0%	49%	51.0%
